# Ethyl 2-phenyl-5,6-dihydro­pyrrolo­[2,1-*a*]isoquinoline-3-carboxyl­ate

**DOI:** 10.1107/S1600536812024853

**Published:** 2012-06-13

**Authors:** Zhao-Peng Yu, Ming Zhao, Cheng-Tao Feng

**Affiliations:** a School of Chemistry and Chemical Engineering, Linyi University, Linyi, Shandong 276005, People’s Republic of China; b Department of Chemistry, Huainan Union University, Huainan 232038, People’s Republic of China; c Department of Chemistry, University of Science and Technology of China, 230026 Hefei, People’s Republic of China

## Abstract

In the title compound, C_21_H_19_NO_2_, the six-membered heterocycle assumes a screw-boat conformation. The phenyl ring is oriented with respect to the pyrrole ring at a dihedral angle of 64.76 (10)°. An intra­molecular C—H⋯O hydrogen bond helps to stabilize the mol­ecular structure. There are weak C—H⋯π inter­actions between inversion-related mol­ecules in the crystal.

## Related literature

For background and applications of lamellarins, see: Bailly (2004 ▶); Zou *et al.* (2011 ▶). For a related compound, see: Feng *et al.* (2012 ▶).
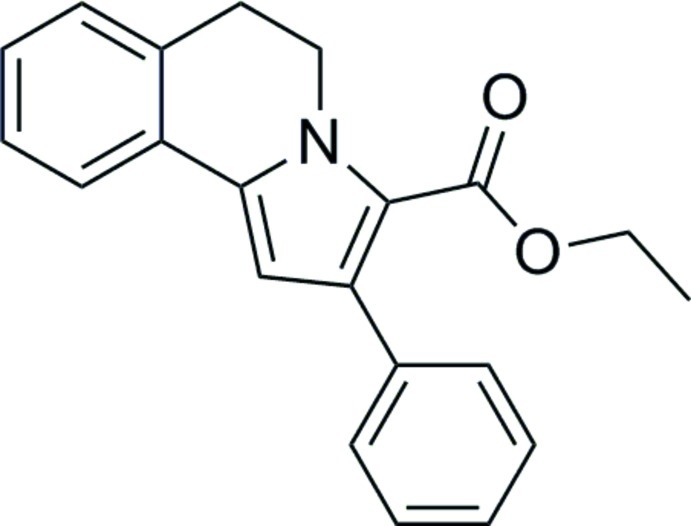


## Experimental

### 

#### Crystal data

C_21_H_19_NO_2_
*M* *_r_* = 317.37
Triclinic, 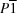

*a* = 8.1527 (6) Å
*b* = 8.4029 (6) Å
*c* = 12.4220 (8) Å
α = 100.117 (6)°
β = 101.155 (5)°
γ = 94.312 (6)°
*V* = 816.66 (10) Å^3^
*Z* = 2
Mo *K*α radiation
μ = 0.08 mm^−1^
*T* = 291 K
0.42 × 0.37 × 0.32 mm

#### Data collection

Oxford Diffraction Gemini S Ultra diffractometer
6799 measured reflections
3340 independent reflections
2233 reflections with *I* > 2σ(*I*)
*R*_int_ = 0.027

#### Refinement

*R*[*F* ^2^ > 2σ(*F* ^2^)] = 0.047
*wR*(*F* ^2^) = 0.117
*S* = 1.02
3340 reflections
218 parameters
H-atom parameters constrained
Δρ_max_ = 0.14 e Å^−3^
Δρ_min_ = −0.18 e Å^−3^

### 

Data collection: *CrysAlis PRO* (Oxford Diffraction, 2007 ▶); cell refinement: *CrysAlis PRO*; data reduction: *CrysAlis PRO*; program(s) used to solve structure: *SHELXS97* (Sheldrick, 2008 ▶); program(s) used to refine structure: *SHELXL97* (Sheldrick, 2008 ▶); molecular graphics: *ORTEP-3 for Windows* (Farrugia, 1997 ▶); software used to prepare material for publication: *SHELXL97*.

## Supplementary Material

Crystal structure: contains datablock(s) I, global. DOI: 10.1107/S1600536812024853/xu5552sup1.cif

Structure factors: contains datablock(s) I. DOI: 10.1107/S1600536812024853/xu5552Isup2.hkl

Supplementary material file. DOI: 10.1107/S1600536812024853/xu5552Isup3.cml

Additional supplementary materials:  crystallographic information; 3D view; checkCIF report

## Figures and Tables

**Table 1:** Hydrogen-bond geometry (Å, °) *Cg*1 is the centroid of the pyrrole ring.

*D*—H⋯*A*	*D*—H	H⋯*A*	*D*⋯*A*	*D*—H⋯*A*
C8—H8*A*⋯O1	0.97	2.29	2.913 (2)	121
C8—H8*B*⋯*Cg*1^i^	0.97	2.69	3.6411 (19)	166

Symmetry code: (i) 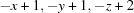.

**Fig. 1.**
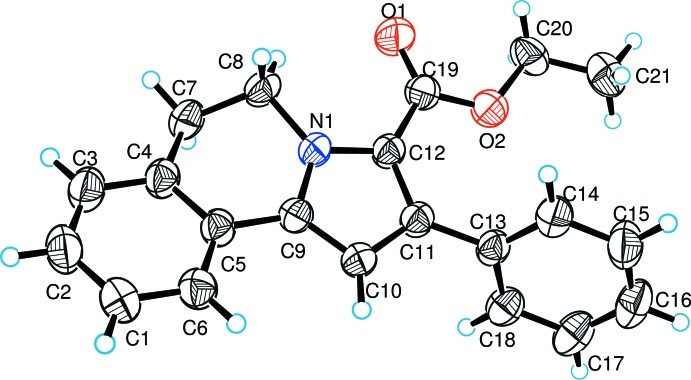
Molecular structure of the title compound with displacement ellipsoids drawn at the 45% probability level.

## supplementary crystallographic information

### Comment

Lamellarin alkaloids, a new family of marine natural products that contain a pyrrolo[2,1-*a*]isoquinoline core, were found to exhibit a wide spectrum of biological activities (Bailly, 2004). Natural as well as synthetic lamellarins should be excellent candidates for the development of new drugs due to their unique skeletal structure and their important biological activities especially as antitumor agents (Zou *et al.*, 2011). As part of our previous studies concerning anticancer agents, we here report a crystal structure of open chain analogues of lamellarins (Feng *et al.*, 2012).

The conformational analysis show that the conformation of 6-membered hetero-ring is screw boat. The phenyl ring is oriented with respect to the pyrrole ring at 64.76 (10)°. An intramolecular C—H···O hydrogen bond helps to stabilize the molecular structure. There is weak C—H···π interaction between inversion-related molecules in the crystal.

### Experimental

Synthesis or separation ??

Colourless blocky single crystals of the title compound suitable for X-ray diffraction analysis were obtained by slow evaporation of the mixed solvent ethanol/CH_2_Cl_2_ (2:1, *v*/*v*) at room temperature for five days.

### Refinement

H atoms were positioned geometrically and allowed to ride on their parent atoms with C—H = 0.93–0.97 Å, *U*_iso_(H) = 1.5*U*_eq_(C) for methyl H atoms and 1.2*U*_eq_(C) for the others.

### Crystal data

**Table:**

C_21_H_19_NO_2_	*Z* = 2
*M**_r_* = 317.37	*F*(000) = 336
Triclinic, *P*1	*D*_x_ = 1.291 Mg m^−^^3^
Hall symbol: -P 1	Mo *K*α radiation, λ = 0.71073 Å
*a* = 8.1527 (6) Å	Cell parameters from 1649 reflections
*b* = 8.4029 (6) Å	θ = 3.3–29.0°
*c* = 12.4220 (8) Å	µ = 0.08 mm^−^^1^
α = 100.117 (6)°	*T* = 291 K
β = 101.155 (5)°	Block, colourless
γ = 94.312 (6)°	0.42 × 0.37 × 0.32 mm
*V* = 816.66 (10) Å^3^	

### Data collection

**Table:**

Oxford Diffraction Gemini S Ultra diffractometer	2233 reflections with *I* > 2σ(*I*)
Radiation source: Enhance (Mo) X-ray Source	*R*_int_ = 0.027
Graphite monochromator	θ_max_ = 26.4°, θ_min_ = 3.3°
Detector resolution: 15.9149 pixels mm^-1^	*h* = −9→10
ω scans	*k* = −10→10
6799 measured reflections	*l* = −15→15
3340 independent reflections	

### Refinement

**Table:**

Refinement on *F*^2^	Primary atom site location: structure-invariant direct methods
Least-squares matrix: full	Secondary atom site location: difference Fourier map
*R*[*F*^2^ > 2σ(*F*^2^)] = 0.047	Hydrogen site location: inferred from neighbouring sites
*wR*(*F*^2^) = 0.117	H-atom parameters constrained
*S* = 1.02	*w* = 1/[σ^2^(*F*_o_^2^) + (0.0468*P*)^2^ + 0.0731*P*] where *P* = (*F*_o_^2^ + 2*F*_c_^2^)/3
3340 reflections	(Δ/σ)_max_ < 0.001
218 parameters	Δρ_max_ = 0.14 e Å^−^^3^
0 restraints	Δρ_min_ = −0.18 e Å^−^^3^

### Special details

**Table:**

Geometry. All e.s.d.'s (except the e.s.d. in the dihedral angle between two l.s. planes) are estimated using the full covariance matrix. The cell e.s.d.'s are taken into account individually in the estimation of e.s.d.'s in distances, angles and torsion angles; correlations between e.s.d.'s in cell parameters are only used when they are defined by crystal symmetry. An approximate (isotropic) treatment of cell e.s.d.'s is used for estimating e.s.d.'s involving l.s. planes.
Refinement. Refinement of *F*^2^ against ALL reflections. The weighted *R*-factor *wR* and goodness of fit *S* are based on *F*^2^, conventional *R*-factors *R* are based on *F*, with *F* set to zero for negative *F*^2^. The threshold expression of *F*^2^ > σ(*F*^2^) is used only for calculating *R*-factors(gt) *etc*. and is not relevant to the choice of reflections for refinement. *R*-factors based on *F*^2^ are statistically about twice as large as those based on *F*, and *R*- factors based on ALL data will be even larger.

### Fractional atomic coordinates and isotropic or equivalent isotropic displacement parameters (Å^2^)

**Table:**

	*x*	*y*	*z*	*U*_iso_*/*U*_eq_	
N1	0.42666 (15)	0.27334 (17)	0.95299 (10)	0.0434 (4)	
O1	0.77604 (15)	0.2297 (2)	1.03663 (11)	0.0740 (5)	
O2	0.69802 (14)	0.16479 (18)	1.18676 (10)	0.0631 (4)	
C5	0.1606 (2)	0.3304 (2)	0.84460 (13)	0.0443 (4)	
C18	0.2738 (2)	0.0419 (2)	1.23460 (15)	0.0539 (5)	
H18	0.2135	−0.0303	1.1714	0.065*	
C8	0.5153 (2)	0.3175 (2)	0.86845 (13)	0.0505 (5)	
H8A	0.6167	0.2637	0.8705	0.061*	
H8B	0.5475	0.4341	0.8840	0.061*	
C12	0.49185 (19)	0.2354 (2)	1.05503 (13)	0.0434 (4)	
C13	0.3623 (2)	0.1813 (2)	1.22237 (13)	0.0454 (4)	
C10	0.2142 (2)	0.2488 (2)	1.03899 (13)	0.0480 (4)	
H10	0.1065	0.2455	1.0537	0.058*	
C11	0.3589 (2)	0.2190 (2)	1.10987 (13)	0.0447 (4)	
C6	0.0014 (2)	0.3795 (2)	0.84292 (14)	0.0536 (5)	
H6	−0.0453	0.3829	0.9058	0.064*	
C1	−0.0877 (2)	0.4233 (2)	0.74877 (15)	0.0600 (5)	
H1	−0.1942	0.4563	0.7482	0.072*	
C9	0.25855 (19)	0.2840 (2)	0.94354 (13)	0.0439 (4)	
C17	0.2729 (3)	0.0075 (3)	1.33909 (17)	0.0671 (6)	
H17	0.2136	−0.0879	1.3458	0.080*	
C4	0.2309 (2)	0.3253 (2)	0.74989 (13)	0.0488 (4)	
C20	0.8689 (2)	0.1447 (3)	1.23534 (15)	0.0621 (5)	
H20B	0.9460	0.2307	1.2238	0.075*	
H20A	0.8983	0.0409	1.2010	0.075*	
C16	0.3596 (3)	0.1139 (3)	1.43305 (17)	0.0696 (6)	
H16	0.3590	0.0908	1.5035	0.084*	
C14	0.4485 (2)	0.2875 (3)	1.31825 (15)	0.0610 (5)	
H14	0.5085	0.3829	1.3120	0.073*	
C15	0.4469 (3)	0.2539 (3)	1.42279 (15)	0.0708 (6)	
H15	0.5052	0.3266	1.4864	0.085*	
C7	0.4011 (2)	0.2665 (2)	0.75443 (13)	0.0556 (5)	
H7A	0.4538	0.3089	0.7001	0.067*	
H7B	0.3873	0.1487	0.7338	0.067*	
C19	0.6685 (2)	0.2107 (2)	1.08874 (14)	0.0474 (4)	
C3	0.1386 (2)	0.3693 (2)	0.65604 (15)	0.0610 (5)	
H3	0.1837	0.3658	0.5925	0.073*	
C2	−0.0192 (3)	0.4181 (3)	0.65534 (16)	0.0646 (5)	
H2	−0.0796	0.4476	0.5917	0.077*	
C21	0.8791 (3)	0.1518 (3)	1.35720 (17)	0.0809 (7)	
H21A	0.8039	0.0649	1.3675	0.121*	
H21B	0.8479	0.2543	1.3900	0.121*	
H21C	0.9921	0.1408	1.3926	0.121*	

### Atomic displacement parameters (Å^2^)

**Table:**

	*U*^11^	*U*^22^	*U*^33^	*U*^12^	*U*^13^	*U*^23^
N1	0.0427 (7)	0.0525 (9)	0.0368 (8)	0.0057 (6)	0.0130 (6)	0.0080 (6)
O1	0.0462 (7)	0.1197 (14)	0.0634 (9)	0.0135 (8)	0.0206 (6)	0.0257 (8)
O2	0.0443 (7)	0.0917 (11)	0.0587 (8)	0.0135 (7)	0.0087 (6)	0.0292 (7)
C5	0.0485 (9)	0.0444 (11)	0.0373 (9)	0.0012 (8)	0.0068 (7)	0.0047 (7)
C18	0.0594 (11)	0.0530 (12)	0.0541 (11)	0.0124 (9)	0.0195 (9)	0.0124 (9)
C8	0.0518 (10)	0.0596 (12)	0.0454 (10)	0.0072 (9)	0.0218 (8)	0.0115 (9)
C12	0.0434 (9)	0.0488 (11)	0.0396 (9)	0.0073 (8)	0.0117 (7)	0.0090 (8)
C13	0.0432 (9)	0.0564 (12)	0.0411 (10)	0.0138 (8)	0.0133 (7)	0.0130 (8)
C10	0.0416 (9)	0.0614 (12)	0.0429 (10)	0.0067 (8)	0.0130 (7)	0.0107 (8)
C11	0.0452 (9)	0.0502 (11)	0.0405 (9)	0.0077 (8)	0.0122 (7)	0.0094 (8)
C6	0.0510 (10)	0.0624 (13)	0.0461 (10)	0.0072 (9)	0.0094 (8)	0.0080 (9)
C1	0.0551 (11)	0.0656 (14)	0.0553 (11)	0.0085 (9)	0.0009 (9)	0.0125 (10)
C9	0.0432 (9)	0.0487 (11)	0.0395 (9)	0.0042 (8)	0.0108 (7)	0.0057 (8)
C17	0.0864 (14)	0.0631 (14)	0.0653 (14)	0.0175 (11)	0.0318 (11)	0.0274 (11)
C4	0.0575 (10)	0.0492 (11)	0.0375 (9)	0.0010 (8)	0.0090 (8)	0.0057 (8)
C20	0.0442 (10)	0.0721 (15)	0.0652 (13)	0.0141 (9)	0.0003 (9)	0.0098 (10)
C16	0.0869 (15)	0.0860 (17)	0.0510 (12)	0.0336 (13)	0.0261 (11)	0.0304 (12)
C14	0.0657 (12)	0.0681 (14)	0.0482 (11)	−0.0001 (10)	0.0126 (9)	0.0115 (10)
C15	0.0806 (14)	0.0864 (18)	0.0420 (11)	0.0104 (12)	0.0088 (10)	0.0075 (11)
C7	0.0629 (11)	0.0663 (13)	0.0407 (10)	0.0068 (10)	0.0198 (8)	0.0093 (9)
C19	0.0468 (10)	0.0487 (11)	0.0456 (10)	0.0046 (8)	0.0112 (8)	0.0050 (8)
C3	0.0706 (13)	0.0690 (14)	0.0427 (11)	0.0028 (10)	0.0107 (9)	0.0128 (9)
C2	0.0707 (13)	0.0690 (15)	0.0501 (11)	0.0055 (11)	−0.0030 (10)	0.0193 (10)
C21	0.0661 (13)	0.103 (2)	0.0663 (14)	0.0083 (12)	−0.0041 (10)	0.0175 (13)

### Geometric parameters (Å, º)

**Table:**

N1—C9	1.3639 (19)	C6—H6	0.9300
N1—C12	1.3775 (19)	C1—C2	1.377 (3)
N1—C8	1.4675 (18)	C1—H1	0.9300
O1—C19	1.2036 (19)	C17—C16	1.371 (3)
O2—C19	1.325 (2)	C17—H17	0.9300
O2—C20	1.442 (2)	C4—C3	1.384 (2)
C5—C6	1.389 (2)	C4—C7	1.502 (2)
C5—C4	1.401 (2)	C20—C21	1.490 (2)
C5—C9	1.461 (2)	C20—H20B	0.9700
C18—C13	1.376 (2)	C20—H20A	0.9700
C18—C17	1.380 (2)	C16—C15	1.367 (3)
C18—H18	0.9300	C16—H16	0.9300
C8—C7	1.508 (2)	C14—C15	1.379 (2)
C8—H8A	0.9700	C14—H14	0.9300
C8—H8B	0.9700	C15—H15	0.9300
C12—C11	1.398 (2)	C7—H7A	0.9700
C12—C19	1.461 (2)	C7—H7B	0.9700
C13—C14	1.385 (2)	C3—C2	1.379 (3)
C13—C11	1.482 (2)	C3—H3	0.9300
C10—C9	1.377 (2)	C2—H2	0.9300
C10—C11	1.396 (2)	C21—H21A	0.9600
C10—H10	0.9300	C21—H21B	0.9600
C6—C1	1.376 (2)	C21—H21C	0.9600
			
C9—N1—C12	109.29 (12)	C3—C4—C5	118.70 (17)
C9—N1—C8	121.26 (13)	C3—C4—C7	123.36 (15)
C12—N1—C8	129.05 (13)	C5—C4—C7	117.91 (14)
C19—O2—C20	118.09 (13)	O2—C20—C21	107.19 (15)
C6—C5—C4	119.82 (15)	O2—C20—H20B	110.3
C6—C5—C9	121.65 (14)	C21—C20—H20B	110.3
C4—C5—C9	118.53 (15)	O2—C20—H20A	110.3
C13—C18—C17	121.11 (18)	C21—C20—H20A	110.3
C13—C18—H18	119.4	H20B—C20—H20A	108.5
C17—C18—H18	119.4	C15—C16—C17	119.77 (18)
N1—C8—C7	109.17 (13)	C15—C16—H16	120.1
N1—C8—H8A	109.8	C17—C16—H16	120.1
C7—C8—H8A	109.8	C15—C14—C13	120.98 (19)
N1—C8—H8B	109.8	C15—C14—H14	119.5
C7—C8—H8B	109.8	C13—C14—H14	119.5
H8A—C8—H8B	108.3	C16—C15—C14	120.1 (2)
N1—C12—C11	107.38 (13)	C16—C15—H15	120.0
N1—C12—C19	122.42 (13)	C14—C15—H15	120.0
C11—C12—C19	130.12 (15)	C4—C7—C8	112.81 (14)
C18—C13—C14	117.99 (15)	C4—C7—H7A	109.0
C18—C13—C11	120.63 (16)	C8—C7—H7A	109.0
C14—C13—C11	121.33 (16)	C4—C7—H7B	109.0
C9—C10—C11	108.17 (14)	C8—C7—H7B	109.0
C9—C10—H10	125.9	H7A—C7—H7B	107.8
C11—C10—H10	125.9	O1—C19—O2	123.10 (16)
C10—C11—C12	107.05 (14)	O1—C19—C12	125.80 (16)
C10—C11—C13	124.05 (14)	O2—C19—C12	111.10 (14)
C12—C11—C13	128.89 (14)	C2—C3—C4	121.00 (17)
C1—C6—C5	120.41 (16)	C2—C3—H3	119.5
C1—C6—H6	119.8	C4—C3—H3	119.5
C5—C6—H6	119.8	C1—C2—C3	120.08 (17)
C6—C1—C2	119.99 (18)	C1—C2—H2	120.0
C6—C1—H1	120.0	C3—C2—H2	120.0
C2—C1—H1	120.0	C20—C21—H21A	109.5
N1—C9—C10	108.09 (14)	C20—C21—H21B	109.5
N1—C9—C5	120.21 (13)	H21A—C21—H21B	109.5
C10—C9—C5	131.69 (15)	C20—C21—H21C	109.5
C16—C17—C18	120.0 (2)	H21A—C21—H21C	109.5
C16—C17—H17	120.0	H21B—C21—H21C	109.5
C18—C17—H17	120.0		
			
C9—N1—C8—C7	−36.6 (2)	C4—C5—C9—N1	15.1 (2)
C12—N1—C8—C7	151.50 (17)	C6—C5—C9—C10	13.7 (3)
C9—N1—C12—C11	1.12 (19)	C4—C5—C9—C10	−166.59 (18)
C8—N1—C12—C11	173.80 (16)	C13—C18—C17—C16	0.8 (3)
C9—N1—C12—C19	178.10 (15)	C6—C5—C4—C3	−0.2 (3)
C8—N1—C12—C19	−9.2 (3)	C9—C5—C4—C3	−179.93 (16)
C17—C18—C13—C14	−1.0 (3)	C6—C5—C4—C7	−178.17 (16)
C17—C18—C13—C11	−178.47 (16)	C9—C5—C4—C7	2.1 (2)
C9—C10—C11—C12	−0.5 (2)	C19—O2—C20—C21	160.33 (17)
C9—C10—C11—C13	178.27 (16)	C18—C17—C16—C15	−0.1 (3)
N1—C12—C11—C10	−0.3 (2)	C18—C13—C14—C15	0.5 (3)
C19—C12—C11—C10	−177.02 (18)	C11—C13—C14—C15	177.98 (16)
N1—C12—C11—C13	−179.07 (17)	C17—C16—C15—C14	−0.4 (3)
C19—C12—C11—C13	4.3 (3)	C13—C14—C15—C16	0.2 (3)
C18—C13—C11—C10	63.7 (2)	C3—C4—C7—C8	146.38 (18)
C14—C13—C11—C10	−113.6 (2)	C5—C4—C7—C8	−35.8 (2)
C18—C13—C11—C12	−117.7 (2)	N1—C8—C7—C4	51.0 (2)
C14—C13—C11—C12	64.9 (3)	C20—O2—C19—O1	3.1 (3)
C4—C5—C6—C1	0.0 (3)	C20—O2—C19—C12	−176.31 (15)
C9—C5—C6—C1	179.69 (17)	N1—C12—C19—O1	4.8 (3)
C5—C6—C1—C2	0.1 (3)	C11—C12—C19—O1	−178.94 (18)
C12—N1—C9—C10	−1.46 (19)	N1—C12—C19—O2	−175.75 (15)
C8—N1—C9—C10	−174.81 (15)	C11—C12—C19—O2	0.5 (3)
C12—N1—C9—C5	177.22 (15)	C5—C4—C3—C2	0.3 (3)
C8—N1—C9—C5	3.9 (2)	C7—C4—C3—C2	178.15 (17)
C11—C10—C9—N1	1.2 (2)	C6—C1—C2—C3	0.0 (3)
C11—C10—C9—C5	−177.24 (18)	C4—C3—C2—C1	−0.2 (3)
C6—C5—C9—N1	−164.60 (16)		

### Hydrogen-bond geometry (Å, º)

Cg1 is the centroid of the pyrrole ring.

**Table:**

*D*—H···*A*	*D*—H	H···*A*	*D*···*A*	*D*—H···*A*
C8—H8*A*···O1	0.97	2.29	2.913 (2)	121
C8—H8*B*···*Cg*1^i^	0.97	2.69	3.6411 (19)	166

Symmetry code: (i) −*x*+1, −*y*+1, −*z*+2.

## References

[bb1] Bailly, C. (2004). *Curr. Med. Chem. Anticancer Agents*, **4**, 363–378. 10.2174/1568011043352939 15281908

[bb2] Farrugia, L. J. (1997). *J. Appl. Cryst.* **30**, 565.

[bb3] Feng, C.-T., Wang, L.-D., Yan, Y.-G., Liu, J. & Li, S.-H. (2012). *Med. Chem. Res.* **21**, 315–320.

[bb4] Oxford Diffraction (2007). *CrysAlis PRO* Oxford Diffraction Ltd, Abingdon, Oxfordshire, England.

[bb5] Sheldrick, G. M. (2008). *Acta Cryst.* A**64**, 112–122. 10.1107/S0108767307043930 18156677

[bb6] Zou, Y.-Q., Lu, L.-Q., Fu, L., Chang, N.-J., Rong, J., Chen, J.-R. & Xiao, W.-J. (2011). *Angew. Chem. Int. Ed.* **50**, 7171–7175. 10.1002/anie.201102306 21698733

